# Defining the concept of reserve in the motor domain: a systematic review

**DOI:** 10.3389/fnins.2024.1403065

**Published:** 2024-04-30

**Authors:** Andreina Giustiniani, Angelo Quartarone

**Affiliations:** IRCCS Centro Neurolesi “Bonino-Pulejo”, Messina, Italy

**Keywords:** motor system reserve, cerebellar reserve, motor rehabilitation, motor unit reserve, motor impairment, compensatory processes

## Abstract

A reserve in the motor domain may underlie the capacity exhibited by some patients to maintain motor functionality in the face of a certain level of disease. This form of “motor reserve” (MR) could include cortical, cerebellar, and muscular processes. However, a systematic definition has not been provided yet. Clarifying this concept in healthy individuals and patients would be crucial for implementing prevention strategies and rehabilitation protocols. Due to its wide application in the assessment of motor system functioning, non-invasive brain stimulation (NIBS) may support such definition. Here, studies focusing on reserve in the motor domain and studies using NIBS were revised. Current literature highlights the ability of the motor system to create a reserve and a possible role for NIBS. MR could include several mechanisms occurring in the brain, cerebellum, and muscles, and NIBS may support the understanding of such mechanisms.

## 1 Introduction

The concept of reserve has been proposed to account for the disjunction observed in some patients between a certain degree of brain damage and its clinical manifestation (Chung et al., 2020c). In this regard, the reserve is defined as a mitigator between brain pathology and the manifestation of symptoms. An initial definition of the reserve has been provided by Stern who distinguished between brain reserve (BR), cognitive reserve (CR), and neural reserve (Stern et al., 2023). Recently, researchers have identified novel forms of reserve taking place in the motor system including a motor reserve (MR), a cerebellar reserve (CER) and a motor unit reserve (MUR). As a corollary, many other related concepts and mechanisms have been proposed, such as compensatory processes, brain maintenance, and brain resilience (Stern et al., 2023).

BR refers to the observation that individuals with more structured brains cope better with brain damage due to neural density and brain volume (Stern et al., 2020). BR correlates with the number of neurons and synapses; thus, it is morphological and quantitative. A better BR contributes to higher performances in the motor domain to the extent that any structural change may influence the functional properties of a network (Stern et al., 2020). On the contrary, MR relies not only on structural properties but also on functional processes. Albeit the concept has not been fully addressed yet, the MR has been preliminary defined as an active process explaining the discrepancy between the severity of symptoms exhibited by patients with Parkinson’s disease (PD) and their levels of brain degeneration (Youn et al., 2023). The ability to perform without functional impairment until the damage reaches a critical threshold and the observation that the amount of motor deficits may differ among patients with similar levels of dopamine depletion has been conceptualized as the MR (Youn et al., 2023). Indeed, in some PD patients, motor symptoms appear only once 50 to 60% of dopaminergic neurons have been lost, thus suggesting that compensatory processes may take place allowing patients to reach rather normal performances in the face of the disease burden. Interestingly, this ability may extend to motor units in the muscles (Habets et al., 2021). In this line, MUR has been recently identified in patients with spinal muscular atrophy (SMA) during fatiguing motor tasks in terms of an unexpected increase in the amplitude of electromyographic activity immediately before failure and reflecting the recruitment of new motor units considered as a reserve (Habets et al., 2021). In addition to PD and SMA, evidence of a reserve is increasingly being provided also in other pathologies such as multiple sclerosis (Sumowski et al., 2009), traumatic brain injury (Kesler et al., 2003), amyotrophic lateral sclerosis (ALS) (Bede et al., 2021), and spinocerebellar ataxia (SCA) (Siciliano et al., 2022). However, the literature is sparse and studies providing a clear definition of the MR considering possible contributing factors as well as suitable methods for MR estimation are lacking. CER has been conceptualized as the capacity of the cerebellum to compensate and restore functions in case of motor damage, tissue damage, or loss of functioning (Kakei et al., 2018; Gelfo and Petrosini, 2022). Such capacity would be allowed by two different mechanisms depending on the etiology. The first mechanism concerns structural changes occurring after focal damage. In this case, the cerebellum may count on the recruitment of novel intact cerebellar areas to compensate (Mitoma et al., 2022). On the contrary, in case of progressive degeneration of cerebellar cells, the cerebellum may induce in the damaged areas a functional compensation based on the avoidance of cell death and the induction of neuroplasticity. Both structural changes and functional processes would be possible due to the presence of a cerebellar reserve. Interestingly, CER would be enhanced by life experiences through neuroplasticity (Kakei et al., 2018) which exerts a neuroprotective role on the cerebellum itself. Overall, animal studies have highlighted the role that CER may play in cerebellar stroke, cerebellar trauma, and spinocerebellar ataxia (Gelfo and Petrosini, 2022). Thus, the importance of understanding and quantifying the cerebellar reserve in humans is straightforward as it may be used to compensate or restore functions in case of cerebellar disease. Furthermore, in case of cortical damage, adequate levels of CER may support cerebellar compensation for cortical motor deficits. It has been suggested that possible techniques to assess CER are magnetic resonance imaging (MRI) or the evaluation of the integrity of specific cerebellar functions (e.g., predictive motor control and motor learning). Interestingly, recent studies are also highlighting the possible role of non-invasive brain stimulation (NIBS) for the assessment and the potentiation of the cerebellar reserve (Manto, 2023). NIBS has been promoted as a safe and reliable tool for causal validation of theoretical models and modulation of brain activity with extensive application in the motor domain in both healthy individuals and patients (Matsuda et al., 2017; Giustiniani et al., 2019, 2021; Learmonth et al., 2021; Calderone et al., 2024). Recently, transcranial magnetic stimulation (TMS) has been used to probe the bimodal balance recovery theory which suggests that, in case of stroke, contralesional influence changes based on the amount of ipsilesional reserve (i.e., it would be inhibitory when there is a high level of ipsilateral reserve and supportive in case of a low level) (Li et al., 2022). Furthermore, TMS has been used to study populations of inhibitory and excitatory interneurons of various motor and non-motor cortical regions within and across cerebral hemispheres (Reis et al., 2008). These physiological measurements have enabled the study of the reorganizational changes in the motor network after brain pathology and may be exploited to understand the relationship between this reorganization and the motor reserve. Furthermore, as a therapeutic tool, TMS has also been used to safely enhance motor performance in many pathological conditions and to study the way by which rehabilitation interventions interact with brain plasticity (Bashir et al., 2010). Therefore, if combined with behavioral and neuroimaging techniques, TMS can contribute to the development of the novel concept of MR by assessing the neural mechanisms underlying this reserve in both healthy individuals and patients. For instance, in patients with motor system pathologies such as PD, stroke, and multiple sclerosis, CER, MUR, and MR may be exploited to delay disease onset, slow progression, and predict individuals’ prognosis. However, despite the potential impact of these reserves, little information is available on their characteristics and development, and their quantification remains an open issue. There is agreement about the importance of physical exercise in their building. However, other life experiences, such as occupation and leisure activities, may contribute. Behavioral proxies, neural substrates, and biological markers underlying MR should be clarified, and a standardized procedure to quantify this reserve should be defined. Such definitions could potentially inform strategies for preventive care and rehabilitation and overall enhance the quality of life of patients and healthy individuals.

Hence, the present study reviews previous literature about reserve in the motor system at different levels. We will provide an overview of studies in the domain of motor, cerebellar, and motor unit reserve, respectively, as well as on motor compensatory processes to address the ability of the brain to create a general form of motor system reserve including all the above-mentioned ones. Moreover, a possible role of NIBS will be discussed.

## 2 Methods

This study was conducted following the Preferred Reporting Items for Systematic Reviews and Meta-Analyses (PRISMA). As this review included a few non-interventional studies focusing on different types of reserve regardless of sample characteristics, we adopted the SPIDER approach (Cooke et al., 2012), for example, our sample included both patients and healthy individuals (S); the observed phenomena were motor reserve, cerebellar reserve, motor unit reserve, brain motor reserve, and compensatory processes (P). Due to the novelty of the concept, the published literature of any research design was considered (D). Neuroimaging, behavioral data, and any other technique used to quantify the reserve were considered as evaluation (E); finally, with respect to research type, we included qualitative, quantitative, and mixed method studies (R).

A review of the studies published from 2000 to 2023 was conducted through a search in the PubMed, Scopus, and Embase databases. The following keywords were used: “motor reserve,” “cerebellar reserve,” “brain reserve,” “motor unit reserve,” “brain compensatory processes,” and “muscular compensatory processes.” An additional search was conducted with the following keywords: “motor reserve,” “cerebellar reserve,” and “transcranial magnetic stimulation” (TMS) and “transcranial direct current stimulation.” The terms were combined using appropriate Boolean operators for search. To be included, studies were required to meet the following criteria:

- Assessing motor, cerebellar, and motor unit reserve/assessing cerebellar and motor reserve using TMS and tDCS;
- Studies assessing brain reserve were included only when brain reserve was linked to the motor domain.

**Figure 1:**
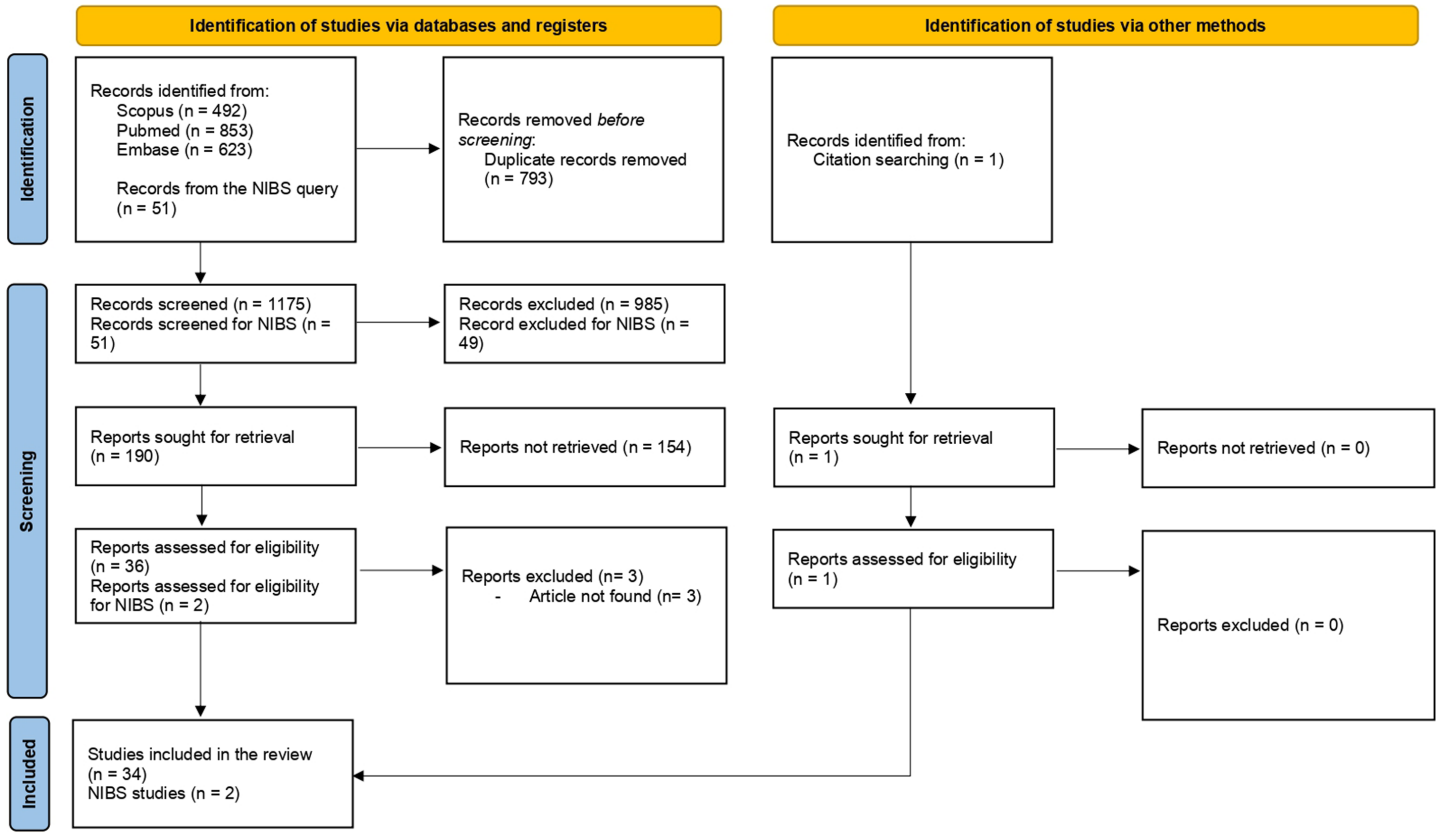
PRISMA flowchart of search method.

Candidate studies were excluded when they were published in non-scientific journals or were not conducted on humans and in case of full-text unavailability (Figure 1).

**Figure 2:**
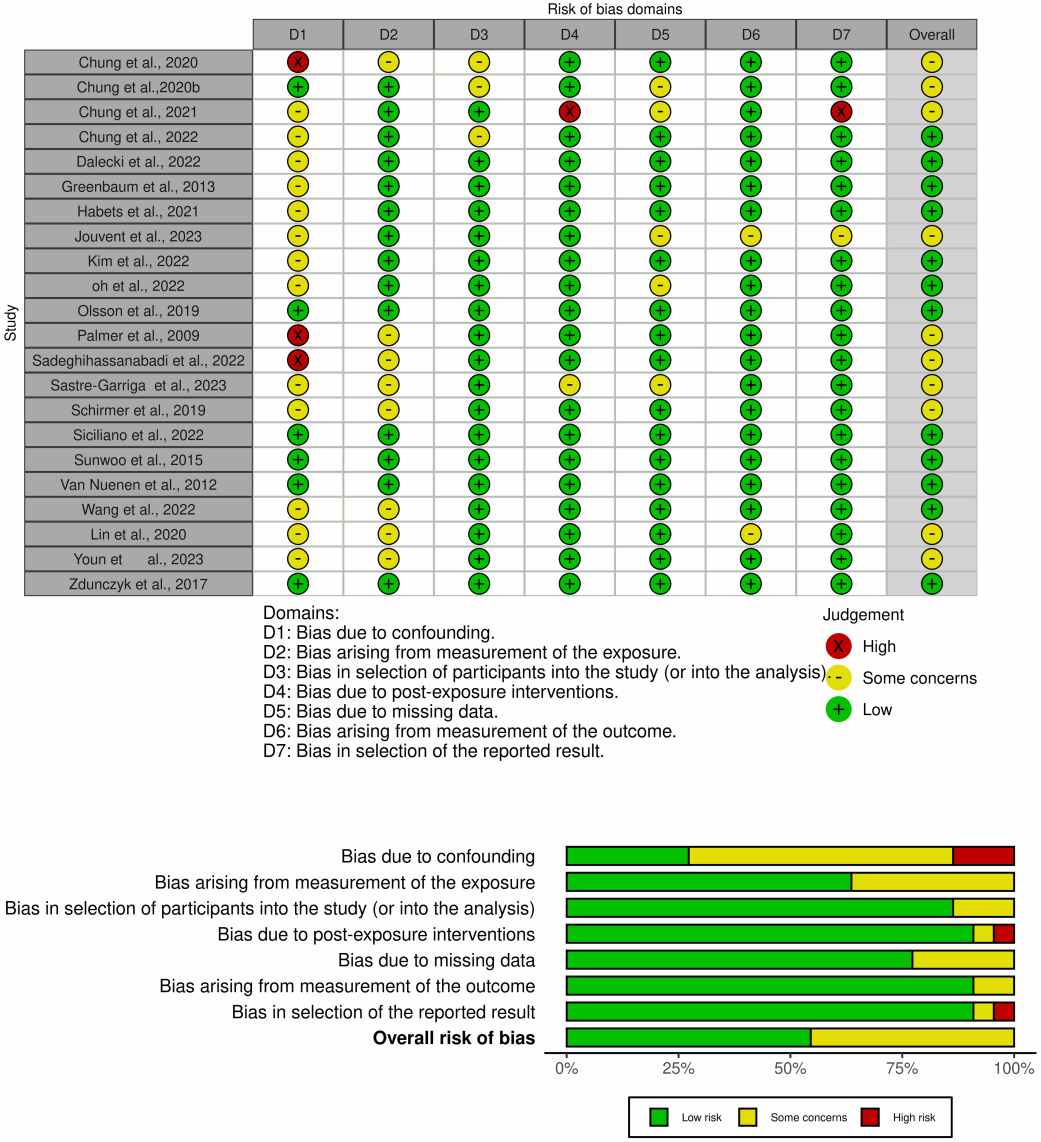
Risk-of-bias graph and study summary review authors’ judgments presented as percentages across all included studies.

The methodological quality of the included studies was assessed using the ROBIN-E tool (Deeks et al., 2011). Each study was rated for seven potential sources of bias: bias due to confounding; bias arising from the measurement of the exposure; bias in the selection of participants into the study; bias due to post-exposure intervention; bias due to missing data; bias arising from the measurement of the outcome; bias in the selection of the reported results. Studies were classified for each domain as having a “low” or “high” risk of bias, respectively, or with “some concerns” (Figure 2).

## 3 Results

Of the initially identified 1968 studies, 34 studies remained as meeting all the inclusion criteria. Among these studies, 11 were reviews (Kakei et al., 2018; Van Loenhoud et al., 2018; Chung et al., 2020c; Bede et al., 2021; Manto et al., 2021; Bastos and Barbosa, 2022; Chung et al., 2022; Gelfo and Petrosini, 2022; Mitoma et al., 2022; Gelfo et al., 2023; Hoenig et al., 2023), one study was a consensus paper (Mitoma et al., 2020), and one study was a letter to the editor (Manto, 2023). Review articles underwent a full-text reading to search for possible eligible papers. Among the remaining 21 studies, 12 focused on MR (Palmer et al., 2009; Sunwoo et al., 2017; Dalecki et al., 2019; Olsson et al., 2020; Chung et al., 2020a,b, 2021, 2022; Kim et al., 2022; Oh et al., 2022; Siciliano et al., 2022; Youn et al., 2023), five studies focused on BR (Jouvent et al., 2016; Sumowski et al., 2016; Schirmer et al., 2019; Wang et al., 2022; Sastre-Garriga et al., 2023), one study investigated CER (Sadeghihassanabadi et al., 2022), one study explored MUR (Habets et al., 2021), and two studies were conducted on compensatory processes (van Nuenen et al., 2012; Greenbaum et al., 2013) (Table 1). The search for NIBS and reserve restituted 51 items. Two articles were included in the review as they met the inclusion criteria (Zdunczyk et al., 2018; Lin et al., 2020).

**Table 1:** Characteristics of the included studies.

**Study**	**Sample size**	**Clinical population**	**Age**	**Gender**	**Type of reserve**	**Reserve estimation**	**Outcome**	**Results**	**Measure of reserve**
Chung et al. (2020a)	134	PD	69.88 ± 8.88	46 females	MR	Relationship between motor deficits and striatal dopamine depletion	Functional brain network and UPDRS scores	A motor reserve network was identified in the basal ganglia, inferior frontal cortex, insula, and cerebellar vermis. Increased degree of functional connectivity in this network was associated with higher reserve. The higher the MR the slower the increase in levodopa equivalent dose in time	Attempts for a direct measure of motor reserve
Chung et al. (2020b)	205	PD	63.78 ± 9.82	48.3% females	MR	Relationship between UPDRS, age, disease duration, and DAT	UPDRS and DAT	Greater MR estimates were associated with a lower risk for levodopa-induced dyskinesia and freezing of gait. Patients with high MR received lower levodopa equivalent dose	Direct assessment of motor reserve
Chung et al. (2021)	408	PD	Not reported	Not reported	MR	Relationship between glucocerebrosidase variants and motor impairment	DAT, GBA, and UPDRS	PD patients with GBA mutations had higher UPDRS scores for the less affected side than those without mutations. The UPDRS sub-scores of the more affected side did not differ between the two PD groups	Not direct assessment of motor reserve
Chung et al. (2022)	163	PD	70.4 ± 8.6	88 females	MR	Relationship between UPDRS scores and DAT availability and disease duration	Correlation between cognitive composite scores and motor reserve estimates and fractional anisotropy	The MR was correlated with verbal memory and years of education. Further fractional anisotropy in the left fornix correlated with MR	Attempts for a direct measure of motor reserve
Dalecki et al. (2019)	64 (and 62 controls)	Sport-related concussion	13.14 ± 1.68 (11.97 ± 1.87 controls)	30 females (29 females in the controls)	MR	Relationship between sport experience and performance	Cognitive-motor integration task	Youth with a concussion history but greater sport experience may have more skill-related motor “reserve”	Motor reserve is hypothesized to explain observed results
Greenbaum et al. (2013)	28	PD	65.7 ± 7.6	20 males	Motor compensatory mechanisms	Relationship between motor scores and Nigrostriatal degeneration	UPDRS and uptake of the [^123^I] FP-CIT	1 out of 4 genes of SNP (Single nucleotide polymorphism) predict UPDRS scores	Compensatory processes considered as motor reserve
Habets et al. (2021)	70 (and 19 controls)	Spinal muscular atrophy	26.9	38 females (10 female in the controls)	MUR	Changes in EMG during the execution of quantitative endurance shuttle tests	Endurance shuttle test (EST)	The decrease in median frequencies and the increase in amplitude reveal motor unit reserve in individuals with SMA during EST	Attempts for a direct measure of motor reserve
Jouvent et al. (2016)	166	CADASIL	52.2 ± 9.7	51% males	BR	Shape of the central sulcus as proxy for disability after stroke	Rankin scale	The severity of disability is related to the shape of the central sulcus. These results support the concept of a motor reserve that could modulate the clinical severity in patients	Direct measure of brain reserve
Kim et al. (2022)	238	PD	70.58 ± 8.93	124 females	MR	Motor impairment and DAT of the posterior putamen	UPDRS	A motor reserve-associated structural network including the frontal region and cerebellum was identified	MR is inferred from DAT availability and UPDRS scores
Oh et al. (2022)	456 (and 22 controls)	PD patients with a history or no history of premorbid cancer	71.5 ± 9.5 (69.0 ± 3.0 controls)	236 females (11 female in the controls)	MR	Relationship between regional SURV and motor impairment	UPDRS and PET	Groups with premorbid cancer showed lower motor scores despite similar levels of dopamine depletion in the posterior putamen relative to those without neoplasia. These results suggest that premorbid cancer acts as a surrogate for motor reserve in patients with PD	MR is assessed comparing SURV and UPDRS
Olsson et al. (2020)	395.369 (with 197.685 skiers)	Skiers and not skiers	36	149.796 females (74.897 females skiers)	MR	Physical activity effects on PD incidence	Incidence of PD among skiers and not skiers	A physical active lifestyle is associated with reduced risk for PD. This association weakens with time and might be explained by a motor reserve among the physically active	MR is hypothesized by the authors to explain observed results
Palmer et al. (2009)	10 (and 10 controls)	PD	66 ± 8	6 female (7 females in the controls)	MR	Relationship between motor network activation and movement speed	Motor task and invariant spatial feature approach	The activity of the motor network during low speed movements in patients was similar to that of controls at higher speed movements	Not direct assessment of motor reserve
Sadeghihassanabadi et al. (2022)	39	Stroke	70.76 ± 12.50	18 females	CER	Relationship between cerebellar volume and functional outcome	Modified Rankin scale	Larger volumes of cerebellar lobules IV, VI, and VIII are positively correlated with positive outcome	Not direct assessment of cerebellar reserve
Sastre-Garriga et al. (2023)	1747 (and 43 controls)	MS	46.35	73.2% females	BR and spinal cord reserve	Association between SCA area and disability	SCA; MRI; PDDS;SCPF	A larger SCA area may be protective against disability, supporting the existence of SCA reserve	Direct assessment of a spinal canal reserve
Schirmer et al. (2019)	453	Acute ischemic stroke	66.6 ± 4.7 years	36% males	BR	BR is considered as a latent variable based on age, systolic blood pressure, and ICV	Modified Rankin Scale, ICV, blood pressure	Higher reserve is associated with more favorable functional post-stroke outcome and might correspond to an overall better vascular health	Direct measure of brain reserve
Siciliano et al. (2022)	12	SCA	48.3 ± 8.3	7 females	MR	Motor reserve index questionnaire (MRIq)	International Cooperative Ataxia Rating Scale	Functional connectivity within a subnetwork including cerebellar and cerebral areas positively correlated with MRIq scores	Direct assessment of motor reserve
Sumowski et al. (2016)	52	MS	60	39 females	BR	Relationship between MLBG and disability status	ICV and EDSS	Larger MLBG predicts lower risk for progression. Patients with smaller MLBG show worse EDSS change	Direct assessment of the brain reserve
Sunwoo et al. (2017)	102	PD	62.9	50 males	MR	Relationship between striatal dopaminergic activity, motor scores, and premorbid physical activity	PET and UPDRS	Engagement in premorbid exercise acts as a proxy for reserve in the motor domain	MR is inferred from Physical Activity Scale for the Elderly scores and level of striatal dopaminergic activity
Van Nuenen et al. (2012)	11 (and 12 controls)	PD	52 ± 7.8	5 males	Motor compensatory mechanisms	Inhibition of the extrastriate body area and of the dorsal premotor cortex	Motor imagery	Following inhibition of the right extrastriate body area, the posture congruency effect was lost in patients. Inhibition of the left dorsal premotor cortex did not reduce the posture congruency. These findings suggest that the right extrastriate body area plays a compensatory role in PD	Compensatory processes but not motor reserve assessment
Wang et al. (2022)	389	PD	61.3	35% female	BR	Interaction between UPDRS scores and deformation-based morphometry	ADL	Patients with greater brain resources had greater compensatory capacity, which was associated with slower rates of clinical progression	Direct measure of brain reserve
Lin et al. (2020)	24	Stroke	50	Not reported	Reserve	Relationship between IHI measured with TMS and UEFM	UEFM, IHI, DTI	Patients with lower impairment in the UEFM had stronger IHI, and patients more impaired in the UEFM had lower IHI. This would reflect a contralesional reserve that would be modulated by lesion severity	Brain reserve in the motor system explored with TMS
Youn et al. (2023)	193	PD	66.2 ± 9.6	104 female	MR	Discrepancy between the severity of motor symptoms and dopaminergic degeneration	UPDRS; DAT DTI	Fractional anisotropy values of frontal, and temporal lobes, limbic structures, nucleus accumbens, and thalamus were correlated with the MR.	Direct assessment of motor reserve
Zdunczyk et al. (2018)	18	Cervical spondylotic myelopathy	65	8 females	Corticospinal reserve and TMS	RMT; Recruitment curve; CSP	Japanese Orthopedic Association score and TMS parameters	Reduced recruitment curve in patients. In patients with mild symptoms, a compensatory higher activation of non-primary motor areas was found	Not direct assessment of MR

Parkinson’s disease (PD); motor reserve (MR); unified Parkinson’s disease rating scale (UPDRS); dopamine transporter availability (DAT); glucocerebrosidase mutations (GBA); motor unit reserve (MUR); cerebral autosomal dominant arteriopathy with subcortical infarcts and leukoencephalopathy (CADASIL); standardized uptake value ratios (SURV); positron emission topography (PET); cerebellar reserve (CER); multiple sclerosis (MS); spinal cord area (SCA); magnetic resonance imaging (MRI); patients determined disease step (PDDS); spinal cord parenchymal fraction (SCPF); brain reserve (BR); intracranial volume (ICV); maximal lifetime brain growth (MLBG); expanded disability status scale (EDSS); activity of daily living (ADL); upper extremity Fugl-Meyer (UEFM); inter-hemispheric inhibition (IHI); diffusion tensor imaging (DTI); transcranial magnetic stimulation (TMS); resting motor threshold (RMT); cortical silent period (CSP).

### 3.1 Brain reserve

The study of Wang et al. (2022) investigated the relationship between subcortical regions volume and clinical progression in PD patients, reporting that lower volumes were associated with faster deterioration of motor scores.

Schirmer et al. (2019) investigated whether BR was able to predict motor deficit measured with the Modified Rankin scale (MRS) in stroke patients. BR was found to predict patients’ performances and recovery after stroke, thus representing a protective mechanism for functional outcomes.

Jouvent considered the shape of the central sulcus as reflecting motor connections and influencing disability in stroke patients (Olsson et al., 2020). Patients with stroke exhibited an association between disability and the size of the hand knob in the central sulcus. This association was discussed in the context of a motor and BR hypothesis in which the shape of the central sulcus may represent a form of reserve.

Sumowski examined whether larger maximal lifetime brain growth (MLBG), measured with the intracranial volume, may be linked to the level of physical disability progression in patients with multiple sclerosis (Sumowski et al., 2016). The author reported that patients with larger MLBG were at lower risk for disability progression and that MLBG may represent a metric to reduce the risk of disability in MS.

Sastre-Garriga explored the role of the spinal cord (SC) in the diagnosis and prognosis of MS. In particular, the estimation of the spinal canal area (SCA) was considered as a proxy of maximal life SC growth. The authors reported an association between the SCA, motor symptoms, and brain volume. In particular, a larger SCA resulted to be protective against disability. These results were interpreted as supporting the concept of a SC reserve depending on the SC area (Sastre-Garriga et al., 2023).

### 3.2 Cerebellar reserve

Only one study investigated the relationship between cerebellar anatomy and patients’ recovery after stroke measured with the MRS (Sadeghihassanabadi et al., 2022). The authors found a positive association for the total cerebellar volume and different lobules involved in motor functions with the MRS. These findings are interpreted as reflecting a cerebellar reserve improving motor outcomes after brain damage.

### 3.3 Motor reserve

Chung et al. (2020a) estimated MR based on initial motor deficits of PD patients and striatal dopamine depletion and identified an MR network by using MRI. The MR was calculated as the difference between the real and the predicted value of the UPDRS. UPDRS scores were found to be associated with age and disease duration and negatively associated with dopamine transporter availability (DAT) in the putamen. A decrease in functional connectivity between regions of an MR network was associated with a lower MR estimate. This is one of the first studies attempting to directly assess MR. Interestingly, in another study, the authors assessed the link between MR and cognitive functions (Chung et al., 2022). Patients underwent F-FP-CIT PET, brain MRI, and neuropsychological tests. The authors found an association between MR, verbal memory, years of education, and white matter integrity in the fornix.

Olsson et al. (2020) retrieved data on PD patients from the Swedish National Patient Registry to study the risk of PD among participants in Vasaloppet compared to matched non-skiers. The main hypothesis was that individuals with higher levels of physical activity had a lower risk of receiving a diagnosis of PD. Vasaloppet was considered a proxy for physical activity. The authors found that physical activity was associated with a lower incidence of PD. These results are discussed in terms of MR which, however, was not directly assessed.

In the study of Sunwoo et al. (2017), premorbid exercise engagement was found to negatively correlate with levels of dopamine reduction in the striatum, in a group of patients with PD.

Kim estimated MR using the UPDRS and DAT in the posterior putamen in a group of PD patients who underwent F-FP-CIT PET and brain MRI scans (Kim et al., 2022). Connectivity strength within an MR functional network indicated the individual’s capacity to tolerate PD-related pathology. In this study, MR was inferred from the relationship between DAT and UPDRS scores.

In the study of Young et al., PD patients underwent an MR assessment, a DAT scan, and a diffusion tensor imaging (DTI) (Youn et al., 2023). The DTI revealed that values of medial, inferior frontal, temporal lobes, limbic structures, nucleus accumbens, and thalamus correlated with the MR.

Chung et al. (2020a) investigated the influence of initial MR on the long-term prognosis of PD. MR was estimated based on initial motor deficits and striatal dopamine depletion by using a residual-based approach. The risk of developing levodopa-induced symptoms was assessed and monitored for a 3-year period. The authors reported that greater MR estimates were associated with lower levodopa-induced symptoms and an overall lower dose of levodopa.

In another study, the same authors investigated the role of glucocerebrosidase (GBA) variants as potential determinants of MR in PD (Chung et al., 2021). Patients underwent a DAT scan and motor assessment by UPDRS. Patients were divided into two groups based on the presence or not of the GBA mutation. DAT availability in the putamen was considered as the proxy for MR. PD patients with GBA mutation had higher UPDRS scores. The GBA variant was found to have a detrimental impact on individual capacity to cope with PD, that is, it has a detrimental effect on MR.

Palmer investigated whether the compensation for motor deficits exhibited by some PD patients depended on changes in the amplitude, or on the spatial extent of activity within brain networks, or on the recruitment of novel regions (Palmer et al., 2009) to successfully complete a visually guided sinusoidal force task. The authors found that healthy subjects exhibited an increase in activity within the striato-thalamo-cortical and the cerebello-thalamo-cortical regions with increasing movement speed during the motor task. Activity at lower speeds in PD patients was found to be similar to that of healthy controls at higher speeds. The authors concluded that PD patients use MR to increase the spatial extent of activation to maintain a near-to-normal performance.

Oh et al. investigated whether cancer history prior to PD diagnosis can enhance MR by assessing the association between motor deficits, measured with the UPDRS, and striatal dopamine depletion (Oh et al., 2022). Depending on the type of tumor, patients were divided into three groups (i.e., no prior neoplasia, premorbid cancerous condition, and premorbid malignant cancer). Each group underwent MRI, PET, and the regional standardized uptake value ratios (SUVRs). In the group with prior neoplasia, the UPDRS score was negatively correlated with SUVRs in the putamen, globus pallidus, thalamus, and ventral striatum, respectively. The premorbid malignant cancer group exhibited lower UPDRS scores than those with no prior neoplasia. The authors concluded that patients with cancer prior to PD diagnosis were less impaired. These results are discussed in terms of an enhancement of the MR by the presence of a premorbid cancer.

Siciliano et al. (2022) investigated whether MR affects motor symptom severity, cognitive functioning, and functional brain networks in patients with spinocerebellar ataxia. MR was assessed using an MR questionnaire including six sections: domestic activities, walking, leisure, working activities, physical exercise, and caring. Scores in the MR questionnaire were found to correlate with the severity of motor symptoms. Functional connectivity patterns in both the cerebellar and the cerebral cortex were found to correlate with MR. This is the only study assessing MR using an *ad hoc* questionnaire and correlating its scores with brain activity.

Dalecki et al. (2019) investigated factors influencing skilled performance recovery in youth with concussions. Sports youths with a concussion history and matched healthy controls were asked to perform eye–hand coordination tasks. Individuals with higher amounts of sport experience reached a performance level matching that of normal participants. The authors conclude that individuals with more sport experiences are able to use compensatory processes in the framework of an MR hypothesis.

### 3.4 Motor unit reserve

Habets et al. (2021) investigated MUR by recording EMG of upper and lower extremities in seven SMA patients during an endurance shuttle test. The authors found a specific pattern of changes characterizing patients compared to controls, that is, a decrease in median frequencies and increasing amplitudes. These changes in EMG activity were interpreted as reflecting a MUR.

### 3.5 Compensatory processes

Greenbaum investigated genetic variants associated with the severity of motor symptoms in PD patients (Greenbaum et al., 2013). In particular, the main hypothesis was that, considering patients with similar levels of striatal terminal degeneration, if genetic variants are associated with the severity of motor symptoms, these variants should be involved in functional compensatory mechanisms for the dopamine deficit in the striatum. Analysis conducted on the single nucleotide polymorphism (SNP) and UPDRS scores revealed that, among many genes analyzed, only the rs6356 SNP in the tyrosine hydroxylase gene was associated with motor symptoms severity and involved in compensatory processes.

van Nuenen et al. (2012) investigated whether the extrastriate body area (EBA) plays a compensatory role in PD by applying continuous theta burst stimulation over the right occipito-temporal cortex. The effects of this inhibition were tested in terms of corticospinal excitability and cerebral motor function. The latter was assessed with a motor task. The authors found that motor performance was lost in patients with PD after the inhibition of the right EBA. These results suggested that this region might play a compensatory role in PD by supporting functions no longer performed by damaged brain areas.

### 3.6 NIBS and reserve

Two studies were included; the first one focused on the relationship between the affected hemisphere and the contralesional one in chronic stroke patients (Lin et al., 2020). In particular, Lin et al. applied TMS to prove the “bimodal balance recovery,” a model suggesting that contralesional influence after stroke varies based on the amount of ipsilesional reserve so that the influence is supportive when there is a low level of reserve whereas it becomes inhibitory in case of a large reserve. Interhemispheric interplay was assessed by testing interhemispheric inhibition (IHI). Motor impairment was assessed with the upper extremity Fugl-Meyer (UEFM), and corticospinal damage was assessed using DTI. The results showed that patients less impaired in the UEFM had higher IHI whereas patients with more impairment had lower IHI. Of note, in this study, the reserve was conceptualized in the context of structural integrity and neurophysiological potential of residual corticospinal pathways measured with both DTI and TMS. In the second study (Zdunczyk et al., 2018), patients with degenerative cervical myelopathy (DCM) underwent a TMS assessment of motor functioning. The authors report higher activation of non-primary motor areas in patients with mild motor symptoms. On the contrary, patients with severe impairment exhibited higher cortical inhibition. These results were interpreted in terms of the corticospinal reserve, and the authors suggested that TMS might be a useful tool to characterize the pattern of functional reorganization in patients with DCM.

## 4 Discussion

The aim of the present study was to review previous studies investigating the concept of reserve in the motor system. Evidence of the ability of the brain to create a reserve has been extensively provided for cognition; on the contrary, that of a reserve in the motor system represents a novel emerging concept. We found preliminary evidence of a reserve at a cerebral, cerebellar, and muscular level as well as a body of literature exploring compensatory processes and brain reserve. Current research on MR has been mostly conducted in patients with PD, multiple sclerosis, SCA, stroke, and concussion, whereas studies on CER focused on patients with SCA, and MUR was investigated in patients with SMA only. With respect to the use of NIBS, the literature is scarce, with only two studies applying TMS to investigate a form of reserve in patients with stroke. As both tDCS and TMS may potentially induce plastic processes, more studies would be needed to investigate their effectiveness to explore neurophysiological correlates of MR and CER. However, currently, there is not enough evidence on their application.

The concept of MR is attracting growing interest in the field of neurosciences, and it has been mostly investigated in patients with PD in which the presence of a higher level of reserve delays diagnosis and reduces symptoms’ severity. Adaptive changes in the basal ganglia, adjustments in neuronal activity in motor cortical areas and their connections, as well as changes in neurotransmitters have been proposed as putative mechanisms underlying these reductions (Blesa et al., 2017).

The majority of the studies conducted in PD assesses MR by measuring DAT activity mostly in the striatum (Chung et al., 2021; Siciliano et al., 2022), for example, the correlation between DAT activity and motor scores or clinical severity would represent the MR. These studies have identified a possible brain network associated with MR in the bilateral basal ganglia, inferior frontal cortex, insula, and cerebellar vermis (Chung et al., 2020c). Furthermore, increased functional connectivity between the medial frontal cortex and the supplementary motor areas and between different cerebellar regions (Kim et al., 2022) has been reported to correlate with MR. In addition to PD, MR has been explored also in patients with SCA2 and ALS in which the presence of an MR would be suggested by the discrepancy observed between the severity of radiological changes and limited functional impairment, especially in the earlier phases of the disease (Bede et al., 2021; Siciliano et al., 2022). However, objective measures of the disease’s burden cannot be considered as directly reflecting the underlying pathological process as well as they should not be considered as an objective measure of MR.

More studies would be needed to investigate whether MR may modulate the severity of motor symptoms and play a role also in other pathological conditions such as stroke (Cappadona et al., 2023) or multiple sclerosis.

Overall, based on the current literature, we may hypothesize that individuals with higher MR (i) could have a lower risk of developing PD as well as other pathologies and levodopa-induced symptoms; (ii) would need reduced doses of medication for motor symptoms (e.g., dopaminergic medications for PD); (iii) would have better rehabilitation outcomes, for instance after stroke; and (iv) would have better motor performances.

The relationship between MR and the incidence of a given motor disease would come of great interest with respect to prevention strategies as it would imply the existence of different recruitment and compensation capacities, as well as differences in neural reorganization to face a certain disease, in individuals with different life backgrounds. For instance, a recent study reported lower PD incidence as well as better motor performances in individuals with higher premorbid exercise activity. Of note, these authors considered physical activity as MR (Olsson et al., 2020). Albeit important, these results should be replicated and confirmed by studies based on a direct assessment of MR rather than on the measure of physical exercise. Instead, agreement is lacking concerning the most appropriate measurement of MR. Although the correlation between DAT and clinical severity may be a first attempt to assess MR in PD patients, this measure could not be similarly adequate to obtain an accurate measure of MR in other pathologies or healthy individuals. In other studies, MR has been instead assessed either by applying custom-made questionnaires (Siciliano et al., 2022) or by assessing years of exercise (Dalecki et al., 2019). However, besides some important recent attempts to assess the behavioral component of MR (Pucci et al., 2023), a comprehensive (i.e., including possible neural correlates and biomarkers) assessment has not been conducted so far.

**Figure 3:**
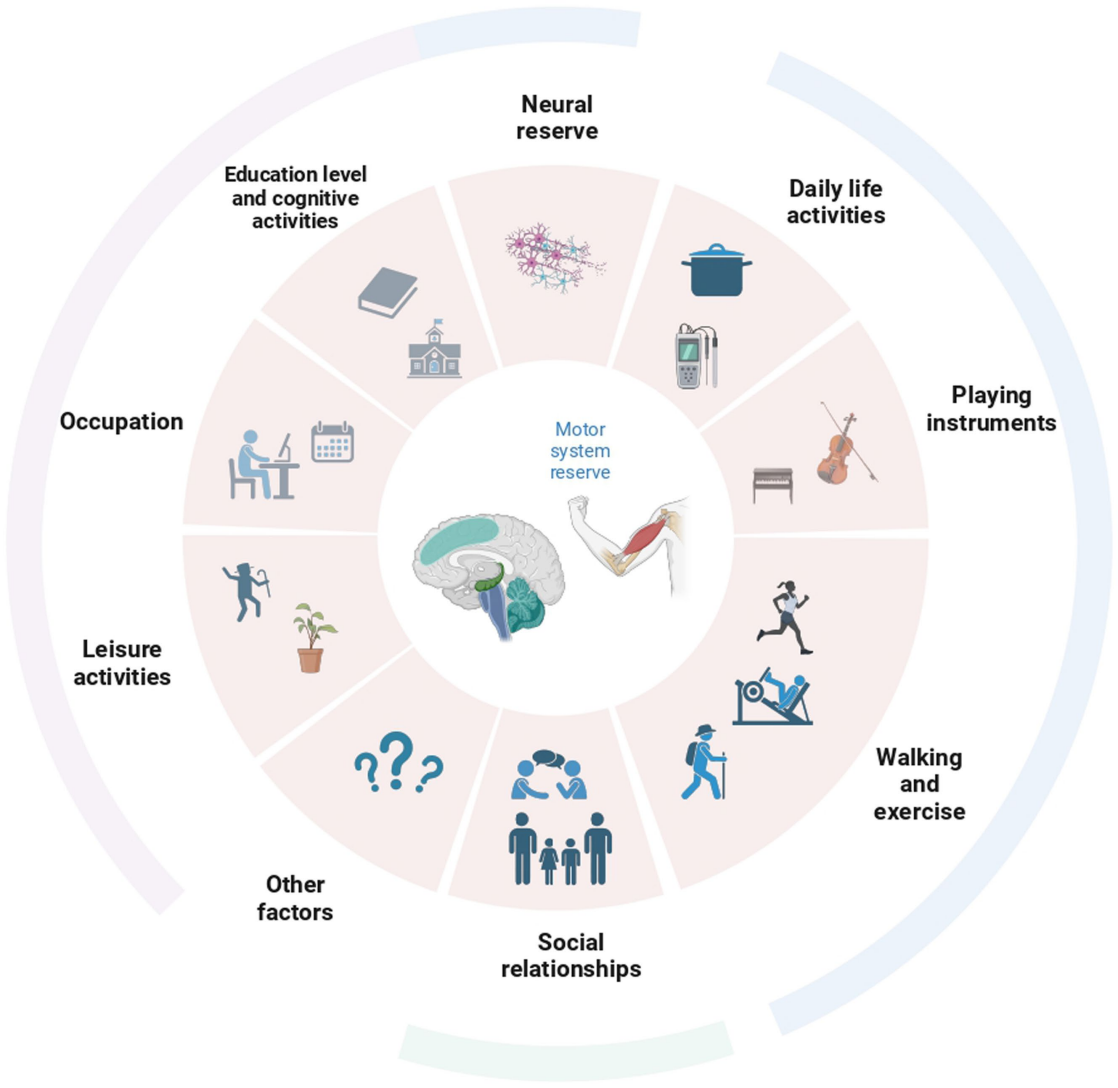
Overview of all the factors possibly contributing to the creation of the reserve in the motor domain. Behavioral assessments in individuals should be conducted taking into account most of these factors.

As it has been done for cognitive reserve, clarifying which factors may contribute to the development of the MR may be a first step toward the definition of a consistent assessment of it. Physical activity may be among the first candidate factors. The practice of long-term exercise reduces the incidence of motor deficits and the risk of a diagnosis of PD (Mak et al., 2017), probably also due to brain changes associated with the training (Calmels, 2020). On the other hand, other factors, such as leisure activities, may contribute (Figure 3). For instance, playing an instrument or dancing induces structural changes in the cerebellum (Abdul-Kareem et al., 2011; Calmels, 2020). Other factors associated with an increased ability to cope with pathology are dominant side laterality and educational levels as well as overall cognitive performances and cognitive reserve (Ham et al., 2015; Sunwoo et al., 2017; Chung et al., 2022). In particular, education exerts a protective role by leading to bigger brain volumes, preserving white matter integrity, and inducing plastic changes (Barulli and Stern, 2013). In addition to education, other factors related to wellbeing may have a potential impact on the reserve and lead to more efficient uses of brain networks in both healthy individuals and patients (Barulli and Stern, 2013). Among these, practicing specific physical activities for a prolonged time, participating in group activities, spending time listening to music, cognitive training, and even social and economic status may impact the building of a motor reserve. Similarly, the motor reserve may present also a biological component, that is, being supported by certain (still not known) genetic factors or being improved by the assumption of specific substances for instance through the diet. Future studies would be needed focusing on the multidimensional nature of factors contributing to the building of this reserve.

Similarly, structural changes in the brain may contribute to the reserve; indeed, previous studies showed that BR and SC reserve levels affect motor recovery (Sumowski et al., 2009; Jouvent et al., 2016; Schirmer et al., 2019; Wang et al., 2022; Sastre-Garriga et al., 2023). In this line, BR or a general form of neural reserve may subserve both cognitive and motor reserves. Of note, single factors, such as physical activity, may represent proxies of MR, but its quantification should be multidimensional, that is, it should consider life experiences, as well as structural and functional rearrangements occurring through neuroplasticity. The hypothesis is the existence of a link between proxies of the reserve, differences in brain structures, functional changes, neuroplasticity, and the protective effect in case of functional loss. Therefore, to test the presence of MR, behavioral assessment and measures of motor performances/symptoms are needed. Ideally, these measurements should also be supported by neuroanatomical and neurofunctional investigations.

**Figure 4:**
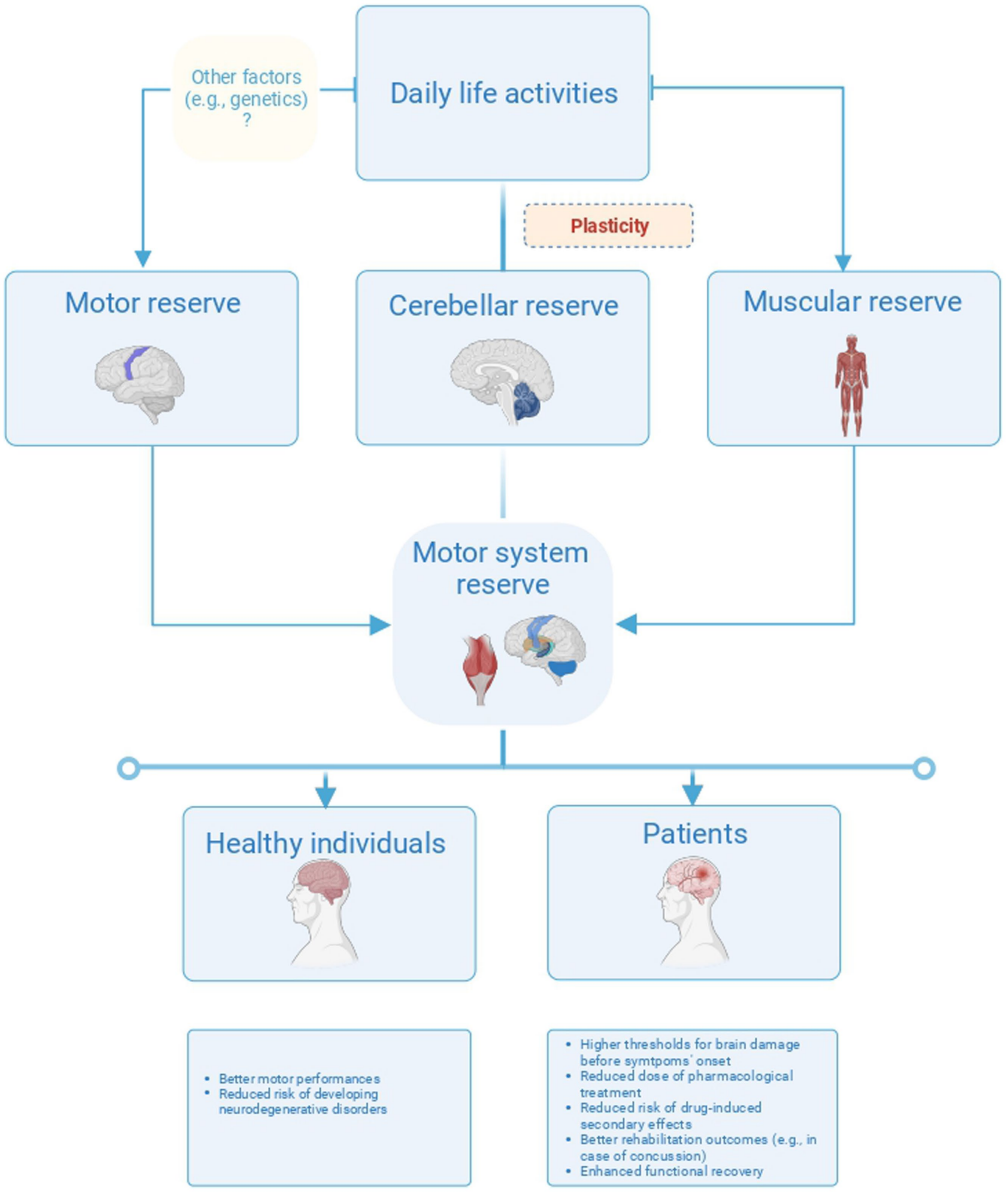
Overview of the three different forms of reserve in the motor system. The figure proposes a model of plasticity-based reserve in the motor system including the motor, cerebellar, and motor unit reserve. These forms of reserves would have different neuroanatomical correlates and different implications for healthy individuals and patients.

We found only a handful of studies investigating CER directly. Attention is growing toward the association between the gray matter volume of the cerebellum and the cognitive reserve (Conti et al., 2021). It would be worthwhile investigating both whether this association also pertains to CER and to what extent CER contributes to motor performances and motor recovery. For instance, the cerebellum exerts a compensative role in motor performances in PD patients through direct connections between the dentate nucleus and the globus pallidus (Wu and Hallett, 2013; Quartarone et al., 2020). These connections could represent a pathway for CER and should be investigated in other motor disorders. In animals, CER has been conceptualized as a mechanism to restore cerebellar output by reorganizing neuron circuits after cerebellar damage (Kakei et al., 2018). This mechanism would arise from the activity of the mossy, the climbing fibers, and the deep cerebellar nuclei, so that reserve would run out in the presence of a significant loss of these cells (Kakei et al., 2018). On the other hand, exposure to environmental enrichment would induce plastic rearrangements (Gelfo et al., 2016) resulting in an improvement of the CER that would exert a protective role in motor abilities (Foti et al., 2011; Gelfo et al., 2016). Unluckily, current literature mostly focused on animal studies; instead, there is a need to define adequate modalities for CER assessment including at least behavioral (e.g., motor and cognitive tasks), biological (e.g., blood and cerebrospinal fluid), and neuroanatomical components (e.g., suitable techniques for CER morphological estimation) to clarify the relationship between functional and structural properties of CER. Afterward, CER, as well as MR, may be assessed in healthy individuals and patients with cerebellar and motor disorders to provide targeted rehabilitation programs taking into account individual level of reserve. For instance, variability in rehabilitation outcomes may depend on the levels of MR or CER whose assessment is often neglected in clinical practice. Instead, assessing the reserve may support the stratification of patients based on the quantification of their residual abilities. Therefore, to provide a comprehensive frame for MR and CER, assessing how life experiences influence individual compensation as well as the correlation between such experiences and prognosis would come of great interest (Figure 4).

Finally, albeit we found only one study exploring MUR, this form of peripheral reserve could interact with reserves in the central nervous system and similarly impact on the individual resilience to motor deficits, for example, the study included in the present review was conducted in patients with SMA and reported that changes in EMG activity during fatiguing motor tasks would reflect MUR (Habets et al., 2021). In particular, MUR could be expressed by an enhancement of EMG activity immediately before task failure reflecting both the recruitment of novel motor units and the increase in the firing rate of the already active ones (Habets et al., 2021). We suggest that exploring the synergy between MUR, MR, and CER would be important to shed light on mechanisms underlying differences in motor performances among individuals and define novel potential therapeutic approaches exploiting a global reserve pertaining to the motor system (De Pasquale et al., 2022).

Two studies investigated compensatory processes in the motor domain focusing on the effect that genetic variants may have on such mechanisms. In particular, genetic variables, such as GBA mutation (Chung et al., 2021) or specific nucleotide polymorphisms (Greenbaum et al., 2013), have been suggested to be responsible for preserved motor performances in PD patients in spite of nigrostriatal dopamine depletion. Compensation has been defined as a set of changes, including the recruitment of supplementary or alternative networks due to dysfunction in the originally employed ones (van Nuenen et al., 2012) that, we suggest, should rely on the presence of an MR. The MR would be built in non-damaged individuals and reflected by their motor performances. Conversely, compensatory processes would pertain to individuals with brain damage or motor disease and reflect the presence of MR. These two studies may have important implications for a definition of MR including also biological factors and could pave the way to future studies on this matter.

Overall, current literature supports the existence of a reserve in the motor domain and suggests that it exerts a protective role for both healthy individuals and patients. The ability to create a reserve pertains, and it is not limited to the central nervous system. However, studies would be needed to explore whether an interaction between MR, CER, and MUR exists and its correlation with the functional and structural properties of the motor system. Furthermore, biological and genetic factors should be taken into account to address differences in MR among individuals.

**Figure 5:**
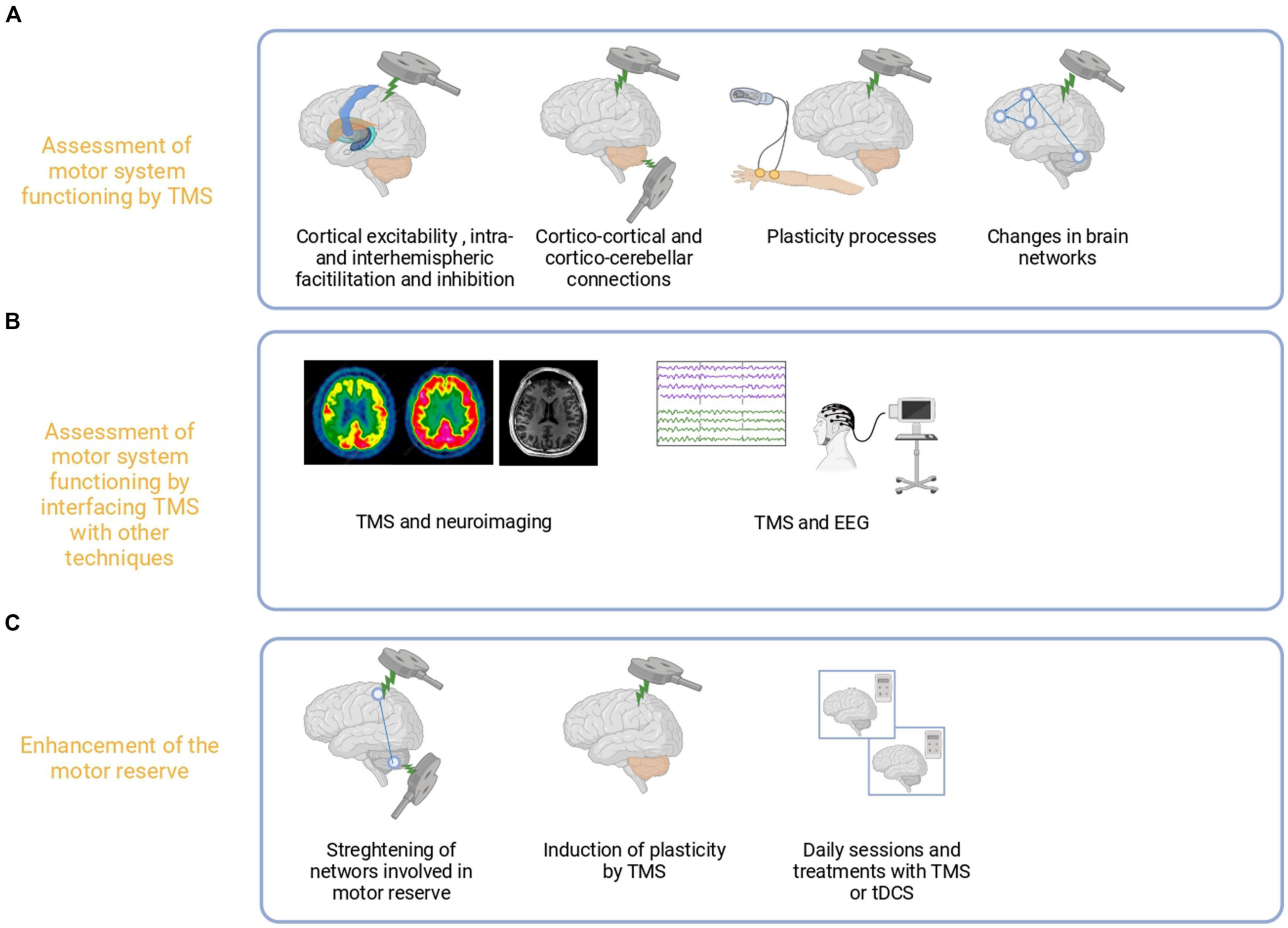
Proposed use of TMS for the assessment and enhancement of the motor reserve. TMS may be used to assess the causal role of motor regions and physiological changes in terms of excitability, plasticity, and connections related to motor reserve **(A)**. Furthermore, TMS may be combined with other techniques such as electroencephalography or MRI to explore MR levels outside primary motor areas **(B)**. Such measures could be combined with behavioral proxies by harnessing and boosting plasticity to improve motor performances in healthy subjects and patients **(C)**.

The current literature does not adequately emphasize the potential role of NIBS in exploring and potentiating the reserve in the motor domain (Manto, 2023). Nevertheless, by definition, the concept of motor reserve cannot neglect the significance of the integrity of the corticospinal pathways in healthy individuals (Lin et al., 2020). In patients, the role of residual structures, intact brain regions, and interhemispheric dynamics within the motor network is crucial, and TMS provides a valuable means to explore it (Figure 5).

For example, numerous studies employing TMS have delved into functional mechanisms or reorganization in patients with motor deficits observing specific patterns of alterations in Motor evoked potentials (MEP) amplitudes, central motor conduction time, cortical silent period, and cortical excitability and plasticity in various different motor diseases (Kojovic et al., 2012; Byblow et al., 2015; Nantes et al., 2016; Hartwigsen and Volz, 2021). While these changes have been suggested as neurophysiological markers associated with motor function recovery or decline, none of these studies have explored the relationship between these markers and the presence of a reserve.

TMS may offer the opportunity to address this gap and explore neurophysiological correlates of MR in the motor system not only in healthy individuals but also in pathological populations. In stroke patients, TMS may support the exploration of the relationship between alteration in interhemispheric inhibition and excitation (Motolese et al., 2023), changes in cortical plasticity and excitability (Byblow et al., 2015), and levels of MR. Indeed, one may assume that the functional reorganization of the corticospinal tract occurring after stroke would be influenced by individuals’ levels of reserve. Similarly, in PD patients, reported alteration in intracortical inhibition, facilitation, and MEP (MacKinnon et al., 2005) may correlate with the presence of a reserve facilitating motor compensatory processes.

Though these hypotheses need to be confirmed by future studies, TMS could support the exploration of the relationship between motor reserve and motor system integrity in various neurological diseases (e.g., multiple sclerosis). Importantly, this may be achieved by applying TMS within the motor system (including the cerebellum) or in other brain regions to understand their role in compensation in case of motor area damage. Further studies are needed to disentangle the relationship between muscular activity and motor unit reserve.

Additionally, especially if combined with neuroimaging techniques, TMS may allow the study of the so-called *perturbation-based biomarkers* reflecting instantaneous brain response to external perturbation (Ozdemir et al., 2021).

For instance, TMS can be employed to monitor the rate of recovery from a state of inhibition, observing changes in cortical excitability within the expected time window following a TMS-induced perturbation. Studies have demonstrated that the recovery time after inhibitory TMS is shorter in individuals with higher levels of resilience (Hall et al., 2020). Supporting this finding, a quicker recovery from continuous theta burst stimulation (TBS) has been reported after moderate exercise (Lowe et al., 2017). If one assumes that exercise is a proxy of the reserve, the time of recovery from TMS-induced perturbation may be predicted by the level of motor reserve connecting the positive effects of physical activity on brain health to patterns of physiological responses to TMS.

Finally, both TMS and tES can be used to enhance MR potentially through neuroplasticity processes. It has been suggested that individual level of cortical plasticity and excitability might represent biomarkers of resilience (Cicchetti and Blender, 2006). This opens the possibility of using NIBS to harness and boost plasticity to potentiate resilience (Passow et al., 2017). Prolonged exposure to stimulation protocols inducing plasticity and network strengthening may be a step toward improving MR directly or by enhancing the effects of its proxies. However, future studies aiming at using NIBS to enhance motor reserve through plasticity should consider and account for the inter- and intra-individual variability in NIBS-induced plastic processes (Huang et al., 2017).

Additionally, differences in individual levels of reserve may account for the lack of response to TMS treatments observed for instance in some stroke patients (Pennisi et al., 1999).

To date, it remains largely unknown what characteristics determine individual response to TMS treatments. Location of the lesion, chronicity, and patients’ age have been proposed as possible factors accounting for differences in current studies (Hoyer and Celnik, 2011). However, these factors alone do not address the issue completely. The risk is a simplified perspective of the complex mechanisms involved in neural reorganization overlooking crucial aspects such as the state of the motor network and the presence of a motor reserve which could be a crucial component to consider in understanding which patients will benefit from TMS treatments (Hartwigsen and Volz, 2021) and traditional rehabilitation.

Overall, albeit we found no studies focusing directly on the application of TMS in MR, current research on TMS application in the motor system can serve as a robust foundation for studies aiming to use NIBS for assessment and potentiation of the motor reserve.

## 5 Conclusion

The current literature supports the emergence of the concept of motor reserve with promising findings. However, we are still far from an exhaustive definition of the concept with several aspects yet to be addressed. Likewise, the precise application of TMS in the context of motor reserve is even further from realization. Understanding the relationship between MR, CER, and MUR, exploiting TMS flexibility, would provide a unique opportunity to take advantage from this precious ability of the motor system for improved motor recovery and treatment outcomes in patients. However, this remains an ambitious yet not fulfilled challenge for neuroscientists.

## Author contributions

AG: Conceptualization, Methodology, Writing – original draft. AQ: Conceptualization, Methodology, Supervision, Validation, Writing – review & editing.
